# Editorial: CRISPR: the game changer in gene and cell therapy

**DOI:** 10.3389/fgene.2024.1517298

**Published:** 2024-12-05

**Authors:** Wenxia He, Meizhu Bai

**Affiliations:** ^1^ Integrated Program for Biological and Genome Sciences, University of North Carolina, Chapel Hill, NC, United States; ^2^ Department of Biochemistry and Biophysics, University of North Carolina, Chapel Hill, NC, United States; ^3^ Department of Genetics, Yale University School of Medicine, New Haven, CT, United States; ^4^ System Biology Institute, Yale University, West Haven, CT, United States; ^5^ Center for Cancer Systems Biology, Yale University, West Haven, CT, United States

**Keywords:** CRISPR, Cas9, gene therapy, cancer therapy, gene editing, genetic screening, CRISPR/Cas9

## 1 Introduction

Gene and cell therapy has undergone transformative advancements since the first successful gene therapy in 1990, which treated a patient with adenosine deaminase deficiency. Over the decades, these therapies have demonstrated immense potential, yet the advent of CRISPR technology has marked a revolutionary shift in the field. CRISPR’s simplicity, precision, and efficiency have reshaped genome engineering, driving the development of innovative therapies for genetic disorders, cancer, and infectious diseases. This editorial highlights the latest advancements in CRISPR technology, discussing its applications and the ongoing challenges in gene and cell therapy, with insights from recent research under the topic CRISPR—The Game Changer.

## 2 CRISPR technology: revolutionizing gene and cell therapy

### 2.1 CRISPR/Cas12a and diagnostics

The introduction of CRISPR/Cas12a has enhanced the precision of genome editing and diagnostic applications. A recent study demonstrated its application in detecting Burkholderia pseudomallei, showcasing CRISPR/Cas12a’s potential for rapid and accurate diagnostics. This advancement underscores CRISPR’s capability to improve early detection and management of infectious diseases, setting a precedent for future diagnostic innovations (Zhang et al.).

### 2.2 Prime editing: precision and versatility

Prime editing represents a significant leap in genetic modification, offering precise insertions, deletions, and replacements without double-strand breaks. Recent research has explored how chromatin structure and sequence context affect prime editing efficiency. These insights are crucial for optimizing this technology, which holds promise for correcting genetic mutations with unprecedented accuracy, thereby advancing therapeutic possibilities for genetic disorders (Kim et al.).

### 2.3 CRISPR/Cas9: disease modeling and therapeutic applications

CRISPR/Cas9 remains a cornerstone of gene editing, with extensive applications in disease modeling and therapy. The technology’s ability to create precise genetic models has accelerated understanding and treatment of various conditions. Innovations in CRISPR/Cas9 applications, including its use in engineering chimeric antigen receptors (CARs) for cancer therapy and modifying hematopoietic stem cells for sickle cell disease, highlight its versatility and impact on clinical outcomes (Kim et al.).

### 2.4 Delivery systems for CRISPR-based therapies

Effective delivery systems are essential for the success of CRISPR-based therapies. Recent advances in viral vectors and extracellular vesicles have improved the precision and safety of delivering CRISPR components. Research into novel delivery methods is crucial for enhancing the efficacy of treatments, particularly in cancer therapies involving CAR-T cells, CAR-NK cells, and tumor-infiltrating lymphocytes (TILs) (Song et al.).

## 3 Current challenges and future directions

Despite its transformative potential, CRISPR technology faces several challenges:

• Safety and Efficacy: Addressing off-target effects and optimizing CRISPR enzymes and guide RNA scaffolds are critical for improving therapeutic safety and efficacy.
• Manufacturing and Delivery: Developing non-viral and viral delivery systems that ensure precise and efficient transport of CRISPR tools remains a significant challenge.
• Ethical and Regulatory Considerations: Navigating the ethical implications and regulatory guidelines for CRISPR-based therapies is essential for their clinical application.

Ongoing research aims to overcome these challenges by refining CRISPR technology, expanding its applications, and improving delivery systems. For instance, advances in CRISPR-based genetic screening are identifying new targets for therapy, while innovations in manufacturing processes are optimizing clinical use.

## 4 Exploring research frontiers in CRISPR-based therapies

As the landscape of CRISPR research continues to expand, several promising avenues have emerged that hold transformative potential for gene and cell therapy. Continued innovation in developing new CRISPR enzymes and guide RNA scaffolds promises to enhance precision in genome editing, addressing the need for greater specificity in therapeutic interventions. Likewise, advancements in CRISPR-based genetic screening are facilitating the discovery of novel gene targets, an essential step toward refining therapies for complex diseases.

Further, the integration of CRISPR technology with cancer immunotherapy, particularly through CAR and TCR-engineered T cells, NK cells, and tumor-infiltrating lymphocytes (TILs), exemplifies CRISPR’s growing impact on personalized cancer treatments. Research into optimizing delivery systems—both viral and non-viral—remains vital, ensuring targeted and efficient administration of CRISPR components in both *in vivo* and *ex vivo* settings. In clinical trials, CRISPR-based therapies continue to navigate challenges and reveal successes, highlighting both the promise and complexity of bringing these therapies to broader patient populations.

The connection between circular RNA and CRISPR is another emerging area, offering insights into new molecular mechanisms and therapeutic applications. Additionally, as CRISPR’s potential to elucidate gene function advances, we are gaining a clearer understanding of disease mechanisms at a molecular level.

## 5 Conclusion

CRISPR technology has ushered in a new era of gene and cell therapy, offering unprecedented precision and potential for treating genetic disorders, cancer, and infectious diseases. As the field continues to evolve, addressing current challenges and embracing innovative research will be key to realizing the full promise of CRISPR-based therapies. We encourage researchers to contribute to this dynamic field, advancing our understanding and application of CRISPR technology to improve patient outcomes and revolutionize medicine.

